# Comparison of statistical methods for the analysis of recurrent adverse events in the presence of non-proportional hazards and unobserved heterogeneity: a simulation study

**DOI:** 10.1186/s12874-021-01475-8

**Published:** 2022-01-20

**Authors:** Noel Patson, Mavuto Mukaka, Lawrence Kazembe, Marinus J. C. Eijkemans, Don Mathanga, Miriam K. Laufer, Tobias Chirwa

**Affiliations:** 1 School of Public Health, University of the Witwatersrand, Johannesburg, South Africa; 2 School of Global and Public Health, Kamuzu University of Health Sciences, Blantyre, Malawi; 3 Mahidol Oxford Tropical Medicine Research unit (MORU), Bangkok, Thailand; 4 Centre for Tropical Medicine, Nuffield Department of Medicine, University of Oxford, Oxford, UK; 5 Department of Biostatistics, University of Namibia, Windhoek, Namibia; 6 Julius Center for Health Sciences and Primary Care, University Medical Center Utrecht, Utrecht University, Utrecht, The Netherlands; 7 Center for Vaccine Development and Global Health, University of Maryland School of Medicine, Baltimore, MD USA

**Keywords:** Recurrent adverse events, Randomised controlled trials, Non-proportional hazards, Unobserved heterogeneity

## Abstract

**Background:**

In preventive drug trials such as intermittent preventive treatment for malaria prevention during pregnancy (IPTp), where there is repeated treatment administration, recurrence of adverse events (AEs) is expected. Challenges in modelling the risk of the AEs include accounting for time-to-AE and within-patient-correlation, beyond the conventional methods. The correlation comes from two sources; (a) individual patient unobserved heterogeneity (i.e. frailty) and (b) the dependence between AEs characterised by time-dependent treatment effects. Potential AE-dependence can be modelled via time-dependent treatment effects, event-specific baseline and event-specific random effect, while heterogeneity can be modelled via subject-specific random effect. Methods that can improve the estimation of both the unobserved heterogeneity and treatment effects can be useful in understanding the evolution of risk of AEs, especially in preventive trials where time-dependent treatment effect is expected.

**Methods:**

Using both a simulation study and the *Chloroquine for Malaria in Pregnancy* (NCT01443130) trial data to demonstrate the application of the models, we investigated whether the lognormal shared frailty models with restricted cubic splines and non-proportional hazards (LSF-NPH) assumption can improve estimates for both frailty variance and treatment effect compared to the conventional inverse Gaussian shared frailty model with proportional hazard (ISF-PH), in the presence of time-dependent treatment effects and unobserved patient heterogeneity. We assessed the bias, precision gain and coverage probability of 95% confidence interval of the frailty variance estimates for the models under varying known unobserved heterogeneity, sample sizes and time-dependent effects.

**Results:**

The ISF-PH model provided a better coverage probability of 95% confidence interval, less bias and less precise frailty variance estimates compared to the LSF-NPH models. The LSF-NPH models yielded unbiased hazard ratio estimates at the expense of imprecision and high mean square error compared to the ISF-PH model.

**Conclusion:**

The choice of the shared frailty model for the recurrent AEs analysis should be driven by the study objective. Using the LSF-NPH models is appropriate if unbiased hazard ratio estimation is of primary interest in the presence of time-dependent treatment effects. However, ISF-PH model is appropriate if unbiased frailty variance estimation is of primary interest.

**Trial registration:**

ClinicalTrials.gov; NCT01443130

## Background

The development of a comprehensive drug safety profile in randomized controlled trials (RCTs) requires statistical methods that can adequately characterize the adverse event (AE) occurrence process (e.g. time of onset, recurrence and duration) [1]. Traditionally, analysis of AEs in IPTp trials, like all clinical trials, is predominantly descriptive and non-parametric such that advanced methods (e.g. parametric and semi-parametric) are rarely used [2–4]. The use of descriptive and non-parametric methods can lead to poor estimates of the effect of treatment on AE recurrence. The descriptive and non-parametric methods may not account for essential aspects of the data structure such as censoring, potential time-varying treatment effect and the AE-dependence. For example, mean cumulative function is the simple and popular non-parametric method used to facilitate the development of trajectories of AEs profiles over the study period, where a patient may experience more than one AEs [5]. However, the method fails to explicitly account for the potential dependence of the AEs or individual patient heterogeneity. The utility of the existing recurrent event methods towards the AEs analysis has been recently considered [6]. However, adopting and interpreting such recurrent events methods results in drug safety assessment setting remains a challenge. This motivates a need for further investigations on the clinical value and practical applicability of the advanced methods [7]. In the framework of benefit-risk assessment, two models (the Andersen Gill model and Prentice, Williams and Peterson model) have been demonstrated to be useful in providing both direct and indirect effects of treatment on AE recurrence [6]. However, these models yield unbiased estimates if the AEs are uncorrelated and do not efficiently capture (i.e. account for) both potential time-dependent treatment effects and unobserved heterogeneity. This motivates the need to consider using flexible parametric shared frailty models that can optimally capture both the time-dependent effects and unobserved heterogeneity, to improve drug safety estimates.

In IPTp clinical trials, the potential of recurrent AEs is high because the preventive antimalarial drugs are repeatedly administered. Recurrent AEs may induce within-patient correlation that originates from two sources: the individual patient unobserved heterogeneity and the dependence between events [8]. The individual patient heterogeneity cannot be explained by the observed/known factors [9, 10], while the between-AE-dependence can be explained based on the observed AEs. The unobserved heterogeneity arises from unmeasured information that partly explains the treatment effect. For example, in IPTp trials, unobserved heterogeneity can arise from the unmeasured inherent characteristics (e.g. physiological changes associated with foetus development). The underlying individual patient-specific unobserved heterogeneity can influence the susceptibility of patients to AE recurrence. In IPTp trials, as the case with some drug trials, the treatment effect depends on follow-up time. Such treatment effect that is a function of follow-up time is called time-varying or time-dependent treatment effect. In the presence of the time-dependent treatment effect, the relative effect of treatment changes over the follow-up time such that the proportional assumption is violated (on log hazard scale). Ignoring the time-dependent effects can lead to biased estimates. Therefore, accounting for the potential time-dependent treatment effects in the analysis of recurrent events is necessary. However, in practice, identifying a model that can account for both unobserved heterogeneity and time-dependent effects can present a challenge. Since the recurrence of AEs can be a reflection of both time-dependent treatment effects and underlying individual patient unobserved heterogeneity. Therefore, it is vital to consider models that account for both issues.

Currently, shared frailty is one of the prevalent models for the analysis of recurrent events. This model can account for both the observed and unobserved heterogeneity. However, the common current applications of the shared frailty model assume that the treatment effect estimate (i.e. hazard ratio) is constant over the follow-up time [11]. In preventive drug trial settings, where there is repeated treatment administration and a long patient follow-up time, the proportional hazard assumption can be invalid when treatment hazard rates, between/across arms, for recurrent events can vary over the follow-up time. For example, in the context of IPTp drug safety assessment, more recurrent AEs are expected close to the day(s) of taking the days. The potential presence of time-dependent treatment effects can pose a challenge in applying the shared frailty model to analyse recurrent events since it becomes complicated to separate time-dependent effects from unobserved heterogeneity [12].

Recent developments in flexible parametric survival models can effectively capture both time-dependent effects and unobserved heterogeneity [13]. Researchers have established, mainly in univariate survival data context, that flexible parametric survival models with restricted cubic splines can accurately model time-dependent effects [14, 15]. However, there is limited literature investigating the efficiency and utility of such methods in the context of multivariate survival data and drug safety assessment. Within multivariate survival data setting, Gasparini et al. recently compared the standard and spline-based shared frailty models under varying heterogeneity and baseline hazard specification assuming proportional hazards [16]. The shared parametric models with splines emerged to be more robust to model misspecification than the standard approaches. However, their work is limited to the context of the proportional hazards assumption. As highlighted above, a challenge arises in applying the shared frailty model when there are time-dependent effects. Using the standard semi-parametric shared frailty models, Balan and Putter demonstrated that the bias of frailty variance estimate, in the presence of time-dependent effects, can be mitigated as the cluster size (i.e. number of recurrent events within an individual) increases [12]. Based on the available literature, it is not clear whether the use of flexible parametric shared frailty with splines models can improve the frailty estimates compared to the standard shared frailty models in the presence of both time-dependent treatment and unobserved heterogeneity.

Our work aims at investigating whether the flexible parametric shared frailty model with non-proportional hazards and restricted cubic splines can improve both the frailty variance and hazard ratio estimates compared to the standard parametric shared frailty models, in the presence of both unobserved heterogeneity and time-dependent treatment effects. The optimal shared frailty model can efficiently capture both unobserved heterogeneity and time-dependent effects. We evaluated the following models; (a) inverse Gaussian shared frailty (ISF-PH) model with proportional hazards and Weibull baseline hazard (b) Lognormal shared frailty model with non-proportional hazard and restricted cubic splines where we model the baseline hazard function with 1 degree of freedom and model the time-dependent treatment effect with 1 degree of freedom (LSF-NPHS1) and (c) Lognormal shared frailty model with non-proportional hazard and restricted cubic splines where we model the baseline hazard function with 3 degrees of freedom and model the time-dependent treatment effect with 1 degree of freedom (LSF-NPHS3). We compare the models using a simulation study assuming an application area of recurrent AEs in IPTp trials. Understanding the statistical properties would enhance the informed choice of statistical methods for the analysis of recurrent AEs. The simulation offers an opportunity to effectively understand the properties of the statistical models since the true data distribution is known, unlike in a practical clinical trial setting. We present the remaining sections of the paper as follows: In the methods section, we provide a detailed review of the models under study, outline the simulation study design and describe the analysis approach. In the results section, we present the findings of our simulation study and apply the models to the analysis of recurrent AEs observed in *Chloroquine for Malaria in pregnancy* trial (NCT01443130). In the discussion section, we discuss the key findings of our work and provide a conclusion.

## Methods

This section outlines notation and provides an overview of the three models that were compared in our simulation study. Specifically, we briefly introduce and discuss the general inferential methodological framework of the models, focussing on model specification, parameter estimation and respective assumptions. The presentation of this section is customized to the context of drug safety assessment in clinical trials. For readers interested in detailed concepts, on recurrent events analysis, they can consult excellent introductory books on recurrent events analysis and frailty models methodology [10, 17, 18], written for a wider audience.

### Notation

We assume that the observation begins at enrolment, denoted as time t = 0. Let *T*_*ik*_ be the total time of the *k*^*th*^ event for the *i*^*th*^ patient where *i* = 1, 2, ………*n*, *k* = 0, 1, 2, ……. *p* and we assume *T*_*i*1_ < *T*_*i*2_ < … < *T*_*ik*_. The AEs are assumed to be recorded over a period of time interval [0, τ] such that the total number of AEs recorded per patient *i* over that time is $=\sum_{k=0}^{\infty }I\left({T}_{ik}\le t\right)$ . The history of the recorded number of events over given time t, denoted as *N*(*t*), 0 ≤ *t*, is defined as a counting process; this forms the basis for easy-to-implement recurrent events analysis. The frailty is defined as *φ*_*i*_ and interpreted as an unobserved covariate common for all recurrent AEs within the patient. Covariate and regression coefficients vectors are defined as ***X***_*ik*_ and ***β*** respectively. The covariates from the covariates vector are considered to be fixed. Under the simulation study design described below, one covariate is considered (i.e. treatment). For the non-proportional hazard models, the treatment effect is allowed to be dependent on time (i.e. ***β***(***t***)). These are further discussed in context in the section below.

### Statistical methods for recurrent events

Recurrent events modelling is popularly based on three main approaches; event counts, event intensity function and joint distribution of gap times between successive events. Event counts-based models are suitable when there are frequent events and event occurrence does not impact the occurrence of the subsequent events [17, 19]. In the context of AEs, some mild AEs may not alter the event process (e.g. mild headache). When events are infrequent and the prediction of the next event is of interest, gap times methods are appropriate [20, 21]. To ensure that the event process history is appropriately incorporated, the intensity function is used; the function defines the instantaneous probability of an event occurring at time t given the event process history [17].

M1 $$\lambda \left(t|H(t)\right)=\underset{\Delta \to 0}{\lim}\frac{\Pr \Big(\Delta N(t)=1\mid \left(H(t)\right)}{\Delta t}$$

Where *∆N(t)* is the number of events in the time interval [t, ∆t), *H(t)* is the history of the event process at time t. Since event counts (i.e. Poisson process) modelling approach assumes that the events are independent such that the event history process at *t*, H(t), does not affect the instantaneous probability of an event at time *t*, the intensity function is reduced to the rate function *ρ(t)* below;

M2 $$\lambda \left(t|H(t)\right)=\underset{\Delta \to 0}{\lim}\frac{\Pr \left(\Delta N(t)=1\right)}{\Delta t}=\rho (t)$$

This implies; ∆*tρ*(*t*) = *E*(∆*N*(*t*)). Hence if we let the expected cumulative rate at time *t* to be μ(*t*), we can derive the expected cumulative rate by integrating the rate function over the interval [0, *t*).

M3 $$\mu (t)={\int}_0^t\rho (s) ds$$

The gap/waiting times method is another alternative approach that focusses on modelling time between consecutive events. Methods based on gap time are suitable mainly in setting where the occurrence of the events is considered relatively infrequent [17]. The work in this paper is confined to multiplicative intensity-based models because it permits deriving and interpretation of failure intensity relative to the treatment under investigation. Shared frailty models are considered as important intensity-based models for the analysis of recurrent events that can account for both measured and unmeasured heterogeneity.

### The shared frailty models

In practice, the common approach to account for unobserved heterogeneity in recurrent events analysis is to use a robust variance (i.e. robust sandwich covariance matrix is used in estimating the log hazard ratio). Adjusting for within-subject unobserved heterogeneity using robust variance has been shown to be inadequate in some settings [22]. One of the solutions to this is explicitly modelling the correlation using patient-specific random effect. Such a model is defined as a frailty model. Frailty models are hazard models having a multiplicative frailty factor [10]; these are conditional models. Overall, the model has frailty, linear predictor and the baseline hazard function as the multiplicative factors. The random effect introduced in frailty models describes the excess risk that cannot be explained by the measured/observed variables [10]. The unmeasured observations may introduce heterogeneity between the analysis units e.g. for recurrent AEs, observations within a patient may be more similar compared to different patients. Hence frailty model attempts to account for the within-patient correlation. Ignoring the potential heterogeneity in modelling recurrent events can lead to underestimation of treatment effect [23, 24].

For recurrent events, when the frailty is modelled as a common random effect for all events within a patient, it is defined as a shared frailty model (see model M4). The dependence accounted for in the shared frailty model is only due to the unobserved individual heterogeneity, *φ*_*i*_. This measure of the extent of heterogeneity among the patients can follow several distributions [18]. Gamma and inverse Gaussian distributions are the common frailty distribution specifications, possibly due to their mathematical tractability and software availability. Across all the patients, the respective random effects are assumed to be independent and identically distributed.

M4 $${\lambda}_{ik}\left({t}_{ij}|{\varphi}_i,\kern0.5em {X}_{ik}\right)={\varphi}_i{\lambda}_0\left({t}_{ij}\right)\mathit{\exp}\left({\boldsymbol{X}}_{\boldsymbol{ik}}^{\mathbf{T}}(t)\boldsymbol{\beta} \right)$$

If the baseline hazard, *λ*_0_(*t*_*ij*_), is unspecified, the model is called a semi-parametric frailty model. Specifying the baseline hazard function ensures that an appropriate shape of the hazard function is captured. However, miss-specification of the baseline hazard distribution may induce bias on regression coefficients [16]. Therefore, caution needs to be exercised on the choice of the baseline hazard distribution by paying attention to the most clinically plausible hazard function. Traditionally, the hazards are assumed proportional over the whole follow-up time. Within the flexible parametric survival methods framework, the proportional hazards assumption can be relaxed to accommodate time-dependent treatment effects (i.e. non-proportional hazards [13]). This can be implemented by interacting treatment with splines of log time as part of the linear predictor. Fitting such flexible non-proportional shared frailty model on log hazard scale is helpful since it can effectively accommodate interpretation and presentation of results. The shared frailty model with non-proportional hazards and restricted cubic splines can be written as;

M5 $$\mathit{\log}\ \left\{{\lambda}_{ik}\left({t}_{ij}|{\varphi}_i,\kern0.5em {X}_{ik}\right)\right\}=s\left\{\log (t)|\gamma, {k}_0\right\}+{\boldsymbol{X}}_{ik}^{\mathbf{T}}\boldsymbol{\beta} +\mathit{\log}\left(\ {\varphi}_i\right)+\sum_{f=1}^Fs\left\{\log (t)|{\delta}_f,{k}_f\right\}{x}_{ik f}$$

The treatment *x*_*ik*_ is interacted with restricted cubic function of log time, *s*{log(*t*)| *γ*, *k*_0_}, such that *γ* is parameter vector and *k*_0_ is knot vector. Similarly, *s*{log(*t*)| *δ*_*f*_, *k*_*f*_} represents a spline function of log time for the *f*^th^ time-dependent effect with parameter vector *δ*_*f*_ and knots vector *k*_*f*_. Since the number of restricted cubic splines can determine the most appropriate model to use we considered to evaluate two flexible parametric shared frailty models as follows;

(i) **LSF-NPHS1:** We considered a shared frailty model assuming lognormal frailty distribution and non-proportional hazards with restricted cubic splines where the baseline hazard function is modelled using 1 degree of freedom and the time-dependent treatment effect is modelled using 1 degree of freedom.
(ii) **LSF-NPHS3**: We also considered a shared frailty model was fitted assuming lognormal frailty distribution and non-proportional hazards with restricted cubic splines where the baseline hazard function is modelled using 3 degrees of freedom and the time-dependent treatment effect is modelled using 1 degree of freedom.

Generally, under shared frailty models, depending on the model specification, parameter estimation can be done using expectation-maximization algorithm, maximum likelihood penalized partial likelihood and Markov chain Monte-Carlo methods [18]. Since in this paper we focus on parametric shared frailty models from a Frequentist perspective, the parameter estimation is based on maximum likelihood estimation. It should be noted that in drug clinical trials not all patients may experience the AEs (i.e. some patients are censored). Censoring in this paper is considered independent of the AE occurrence. Specifically, the model parameters can be estimated by maximizing marginal log-likelihood as shown below; Let *Z* be the vector containing the observed patient-specific information such that

$$Z=\left(\left({y}_{ij},{\xi}_{ij},{x}_{ij}\right)\ \right|j=0,1,\dots k\Big)$$

where *y*_*ij*_, *ξ*_*ij*_ and *x*_*ij*_ are time to AE occurrence, AE occurrence indicator (1 for AE occurrence and 0 otherwise) and fixed covariate for patient *i* and *jth* AE occurrence, respectively. Let ***U*** be a vector containing the latent information (the frailty terms) for n patients.

$$U=\left({u}_1,{u}_2,\dots, {u}_n\right),$$

therefore, the marginal log-likelihood of the observed data *Z* can be written as:

$${l}_{marg}\left(\xi, \beta, Z|\tau \right)=\sum_{i=1}^n\left\{\left[\sum_{j=0}^k{\xi}_{ij}\left(\log \left({h}_0{y}_{ij}\right)\right)+\left({X}_{ij}^T\beta \right)\right]+\log \right[{\left(-1\right)}^{d_i}\left({\mathcal{L}}^{\left({d}_i\right)}\left(\sum_{j=0}^k{H}_0\left({y}_{ij}\right)\exp \left({X}_{ij}^T\beta \right)\Big)\right]-\log \left[\mathcal{L}\left(\sum_{j=0}^k{H}_0\left({\tau}_{ij}\right)\exp \left({X}_{ij}^T\beta \right)\right)\right]\right\}$$

where *d*_*i*_ represents the total number of events for patient and ${\mathcal{L}}^{(v)}(.)$ represents the *v*^*th*^ derivative of the Laplace transform for the derivative. Availability of the Laplace transform simplifies the maximization of the log-likelihood in order to compute the desired model parameters. Under the time-dependent treatment effect, the fixed treatment effect *β* can be replaced by the time-dependent treatment effect *β*(*t*).

### Simulation study

This simulation study was conducted and reported based on “Aims, Data generating process, Method of analysis, Estimands and Performance” approach [25] since it provides a scientifically coherent structured framework for designing, interpreting and reporting simulation studies.

#### Aim

There is a dearth of literature highlighting the performance of recurrent statistical methods when both AE-dependence and heterogeneity is accounted for in analysis of recurrent AEs in clinical trials. The aim of the simulation study was to evaluate whether the use of Lognormal shared frailty model with restricted splines and non-proportional hazards improves both the frailty variance and hazard ratio estimates in the presence of time-dependent effects and unobserved heterogeneity under varying unobserved heterogeneity levels and sample sizes. The performance of this flexible model was compared against the standard proportional hazards-based inverse Gaussian shared frailty model with proportional hazard and Weibull baseline hazard distribution assumptions.

#### Data generating mechanisms

The data generating mechanism (DGM) in this simulation study focussed on the specification and estimation of frailty variance and hazard ratio in the presence of time-dependent treatment effects and unobserved heterogeneity (Table 1). For each scenario, we considered 1000 simulations, lognormal frailty distribution, Weibull baseline hazard, fixed effect treatment effect of log hazard ratio 0.5 and up to a maximum of 4 AEs experienced by a patient. The choice of possible maximum of 4 AEs per patient was aimed at mimicking a setting for moderately high occurrence of recurrent AEs, in IPTp trials. The choice of lognormal frailty distribution was driven by the mathematical tractability of the distribution, availability of software for implementation and interpretability of the results in the context of AEs. The DGMs varied sample size (*n* = 300, *n* = 500), frailty distribution variance (0.25, 0.50, 0.75) and time-dependent effect (0.03, − 0.03). The choice of the sample size was intended to reflect sample sizes that are commonly employed in most IPTp trials. Choice of frailty variance (i.e. a measure of the strength of the unobserved heterogeneity within an individual) intended to represent a setting 0.25, 0.5 and 0.75 represented weak variance, moderate variance and heavy variance respectively. The time-dependent treatment effect allowed the fixed effect treatment effect of log hazard ratio 0.5 to decay over time (when set at − 0.03) or increase over time (when set at 0.03) or remain constant when set at 0.0). All the DGMs were parametrically based, to accommodate the investigation of several scenarios unlike resampling that confines investigation to data with properties only for the application data under study [25]. Hence, we simulated recurrent AE data similar to common scenarios that are practically and theoretically expected in IPTp trials. In all the simulation scenarios, the data generation mimicked a randomized controlled trial with two treatment arms. In all scenarios under study, we assumed that there were no substantial competing risks observed during the trial such that censoring was considered independent. The individual follow-up times were simulated based on uniform distribution and maximum follow-up time of 12 months (i.e. administrative censoring was set at 1 year), using *Survsim* package in stata. The choice of 12 months of follow-up time was motivated by the fact that most IPTp trials follow-up the mother for 12 months from enrolment to beyond delivery.

**Table 1:** Frailty variance estimates bias, coverage probability across shared frailty models in the presence of unobserved heterogeneity and non-proportional hazards

Data Generating Mechanisms				ISF-PH Model	LSF-NPHS1 Model	LSF-NPHS3 Model	% gain in precision relative to ISF-PH model	
Scenario^a^	Sample size	Frailty variance	TDE	Bias (MCSE), Coverage (MCSE), MSE (MCSE)	Bias (MCSE), Coverage (MCSE), MSE (MCSE)	Bias (MCSE), Coverage (MCSE), MSE (MCSE)	LSF-NPHS1% gain in precision (MCSE)	LSF-NPHS3% gain in precision (MCSE)
1	500	0.25	0.03	−0.0389 (0.0064), 96.40 (0.5891), 0.0423 (0.0021)	−0.0556 (0.0060), 95.90 (0.6270), 0.0386 (0.0020)	− 0.0263 (0.0061), 96.20 (0.6046), 0.0374 (0.0019)	14.6985 (0.7269)	11.1644 (1.6768)
2	500	0.50	0.03	−0.0431 (0.0051), 95.00 (0.6892), 0.0278 (0.0012)	−0.1329 (0.0045), 87.10 (1.0600), 0.0375 (0.0015)	−0.1098 (0.0045), 90.20 (0.9402), 0.0323 (0.0014)	30.9883 (0.9472)	27.8901 (1.8680)
3	500	0.75	0.03	− 0.0689 (0.0048), 92.80 (0.8174), 0.0280 (0.0013)	− 0.2151 (0.0041), 61.80 (1.5365), 0.0628 (0.0020)	−0.1939 (0.0041), 69.10 (1.4612), 0.0544 (0.0019)	40.7088 (1.0645)	38.4618 (1.9530)
4	300	0.25	0.03	−0.0363 (0.0079), 97.20 (0.5217), 0.0637 (0.0029)	−0.0542 (0.0073), 97.50 (0.4937), 0.0569 (0.0027)	− 0.0248 (0.0075), 96.70 (0.5649), 0.0561 (0.0027)	12.5407 (1.8701)	12.5407 (1.8701)
5	300	0.50	0.03	−0.0557 (0.0069), 94.90 (0.6957), 0.0504 (0.0025)	−0.1448 (0.0061), 90.60 (0.9228), 0.0575 (0.0027)	− 0.1221 (0.0061), 92.50 (0.8329), 0.0518 (0.0025)	29.3473 (0.9645)	28.3377 (1.8182)
6	300	0.75	0.03	− 0.0674 (0.0062), 94.50 (0.7209), 0.0431 (0.0020)	− 0.2141 (0.0052), 76.60 (1.3388), 0.0729 (0.0026)	− 0.1950 (0.0053), 80.90 (1.2431), 0.0660 (0.0025)	42.0668 (1.1767)	37.7659 (2.0100)
7	500	0.25	− 0.03	− 0.0212 (0.0063), 96.10 (0.6122), 0.0405 (0.0019)	− 0.0694 (0.0060), 95.20 (0.6760), 0.0413 (0.0021)	− 0.0348 (0.0061), 95.90 (0.6270), 0.0384 (0.0020)	9.7913 (0.6757)	7.8448 (1.5502)
8	500	0.50	− 0.03	−0.0317 (0.0051), 95.30 (0.6693), 0.0268 (0.0012)	− 0.1425 (0.0045), 85.60 (1.1102), 0.0405 (0.0016)	− 0.1163 (0.0045), 89.00 (0.9894), 0.0341 (0.0014)	27.3902 (0.9049)	25.1537 (1.7685)
9	500	0.75	−0.03	− 0.0605 (0.0048), 93.30 (0.7906), 0.0269 (0.0013)	− 0.2236 (0.0041), 59.20 (1.5541), 0.0668 (0.0021)	− 0.1998 (0.0041), 67.70(1.4788), 0.0569 (0.0019)	38.1980 (1.0547)	37.1299 (1.8588)
10	300	0.25	−0.03	− 0.0190 (0.0079), 97.00 (0.5394), 0.0621 (0.0027)	− 0.0680 (0.0075), 97.70 (0.4740), 0.0602 (0.0028)	− 0.0335 (0.0075), 97.00 (0.5394), 0.0577 (0.002)	11.1739 (0.7791)	9.1266 (1.7207)
11	300	0.50	−0.03	− 0.0454 (0.0069), 94.80 (0.7021), 0.0493 (0.0024)	− 0.1549 (0.0061), 89.50 (0.9694), 0.0614 (0.0029)	− 0.1286 (0.0061), 92.50 (0.8329), 0.0537 (0.0026)	26.3505 (0.9108)	26.8512 (1.7308)
12	300	0.75	−0.03	−0.0588 (0.0062), 94.80 (0.7021), 0.0418 (0.0019)	− 0.2219 (0.0053), 75.70 (1.3563), 0.0768 (0.0027)	− 0.2005 (0.0053), 79.90 (1.2673), 0.0684 (0.0026)	39.0878 (1.1318)	36.0944 (1.9042)
13	500	0.25	0	−0.0296 (0.0064), 96.50 (0.5812), 0.0412 (0.0020)	− 0.0619 (0.0060), 95.70 (0.6415), 0.0398 (0.0020)	− 0.0300 (0.0061), 95.90 (0.6270), 0.0378 (0.0019)	12.1870 (0.6812)	9.4918 (1.6201)
14	500	0.50	0	−0.0373 (0.0051), 95.00 (0.6892), 0.0272 (0.0012)	− 0.1373 (0.0045), 86.80 (1.0704), 0.0389 (0.0016)	− 0.1127 (0.0045), 89.70 (0.9612), 0.0331 (0.0014)	29.0623 (0.8985)	26.5314 (1.8048)
15	500	0.75	0	−0.0644 (0.0048), 93.00 (0.8068), 0.0274 (0.0013)	−0.2188 (0.0041), 61.30 (1.5402), 0.0646 (0.0020)	−0.1964 (0.0041), 68.80 (1.4651), 0.0554 (0.0019)	39.4216 (1.0400)	37.9288 (1.8940)
16	300	0.25	0	−0.0273 (0.0079), 97.10 (0.5307), 0.0627 (0.0028)	− 0.0605 (0.0074) 97.70 (0.4740), 0.0583 (0.0028)	−0.0283 (0.0075), 96.70 (0.5649), 0.0567 (0.0027)	13.2530 (0.7841)	10.8368 (1.7814)
17	300	0.50	0	−0.0500 (0.0069), 94.80 (0.7021), 0.0495 (0.0024)	−0.1491 (0.0061), 90.20 (0.9402), 0.0591 (0.0028)	− 0.1249 (0.0061), 92.60 (0.8278), 0.0526 (0.0026)	27.7522 (0.9164)	27.0827 (1.7637)
18	300	0.75	0	−0.0627 (0.0062), 94.50 (0.7209) 0.0423 (0.0020)	−0.2174 (0.0052), 76.20 (1.3467), 0.0745 (0.0027)	− 0.1972 (0.0053), 80.60 (1.250) 0.0668 (0.0026)	40.6654 (1.1293)	37.0383 (1.9578)

*TDE* time dependent effect

^a^each scenario is based on 1000 simulations

The hazard functions were simulated using inversion method, under Weibull baseline hazard function, accommodating both time-varying treatment effect and unobserved heterogeneity with lognormal frailty distribution. The inversion method enabled sampling from a given distribution using uniform distribution and quantile function [26] where uniform distribution was assumed to have minimum 0 and maximum of 1. An overview of the investigated scenarios appears in Table 1 below.

During the data generation process, convergence of the models was also continuously monitored. After the data generation descriptive summary statistics were also done to check for any abnormal observations. For each scenario, graphical exploration of the distribution of the estimates was done to ascertain the assumed underlying distribution of the generated data.

### Simulation study targets

Of primary interest in this study was shared frailty models` ability to effectively capture the unobserved heterogeneity. Therefore, the primary estimand of interest was log frailty variance *φ*_*i*_ for each of the investigated models. Since our second interest was to assess the ability of the models in capturing time-dependent treatment effect, the log hazard ratio ***β*** computed at 3 months of follow-up time was considered as a secondary estimand. We opted for log hazard ratio at the specific follow-up time in order to ensure that the estimates from both the proportional and non-proportional hazard models are comparable.

#### Methods for data analysis

Each simulated data set was analysed using the three methods under investigation: ISF-PH, LSF-NPHS1 and LSF-NPHS3. Based on our simulation scenarios, the ISF-PH model miss-specified the proportionality of the hazards although it correctly specified the baseline hazard function (i.e. the baseline hazard function for the ISF-PH was modelled using Weibull distribution). The LSF-NPHS1 and LSF-NPHS3 models correctly specified the proportionality of the hazards although the baseline hazard function is approximated by the restricted cubic splines. When there are non-proportional hazards, reporting of a single average HR is considered not practically useful. Therefore, in order to ensure that the performance measures for the HR estimates are comparable across the three models, we computed hazard ratio at 3 months of follow-up time since enrolment (i.e. LSF-NPHS1 and LSF-NPHS3 are non-proportional hazard models and ISF-PH is a proportional hazard model). The choice of the time at which the HR was calculated was motivated by our practical experience where most patients take approximately half of their scheduled IPTp doses by third month of follow-up time.

The three shared frailty models (ISF-PH, LSF-NPHS1 and LSF-NPHS3) are also used in the analysis of recurrent adverse events that were observed in the *Chloroquine for Malaria Prevention in Pregnancy* trial (NCT01443130) data. The motivating data description, data analysis and results appear in the application section of this paper, after simulation results presentation.

#### Performance measures

In this paper, our model performance measures of interest were defined as described in Morris et al [25]. Bias and MSE were main performance measures for our estimands. For frailty variance estimand, we assessed the model performance based on precision, bias, MSE and coverage probability of 95% confidence interval of the frailty variance estimates. For the hazard ratio estimand, we assessed the model performance based on precision, bias and MSE. Point estimates of performance are reported including Monte Carlo standard errors in order to highlight the certainty of our point estimates.

We used bias in order to quantify whether the estimator of interest targeted the true value of interest. Bias is defined as the difference between the expected value of an estimator and the true parameter value. Secondly, in order to integrate bias and variance of an estimator into one performance measure, we computed the mean square error (MSE). MSE is defined as sum of the squared bias and variance of the estimator. In defining relative precision gain, ISF-PH model was considered as a reference model. Therefore, precision gain is defined as the inverse squared ratio of the empirical SE of the respective flexible parametric model (i.e. LSF-NPHS1 or LSF-NPHS3) to the empirical SE of the ISF-PH model. Additionally, coverage was also computed, defined as proportion of confidence intervals for an estimator that contain the true parameter value.

#### Software and computer hardware specification

The simulation study was conducted using user-written *survsim* and *merlin* packages in Stata version 15.1 [27–29]. The analyses of the simulated and application data were also done using *streg* built-in function in Stata for the ISF-PH model.

The simulation study was performed using the Stata version 15.1 that was installed in a purposely-built Threadripper 3990X GT 1030 workstation, equipped with a Threadripper 3990X (288 MB Cache, 64x Cores, 4.3GHz) turbo processor, MSI TRX40 PRO 10G AMD Ryzen Threadripper Motherboard, Nvidia GeForce RTX 2070 SUPER OC Ed. 8GB GDDR6 GPU, Corsair Vengeance PRO RGB 3200 MHz 64 GB High-Performance Gaming, Hikvision E2000 1 TB M.2 SSD that reads up to 3.5 GB/s and a 4 TB High-Performance Hard Disk Drive (HDD).

### Ethical considerations

The *Chloroquine for malaria in pregnancy* trial was conducted in accordance with the Declaration of Helsinki and Good Clinical Practice guidelines. The trial obtained ethical approval from the Institutional Review Board at the University of Maryland, the College of Medicine Research and Ethics Committee at the University of Malawi and the Malawi Pharmacy Medicines and Poisons Board. The current study was approved by the University of the Witwatersrand’s Human Research Ethics Committee.

## Results

### Frailty variance estimates coverage probability, bias and MSE across the shared frailty models

Based on 1000 simulations for each scenario, we observed a 100% convergence rate of all the models that were fitted. Across all the scenarios, the magnitude of bias for the frailty variance estimates increased as the known unobserved heterogeneity variance increased from 0.25 to 0.75 whether the sample size was 300 or 500 (Table 1). The observed bias across all the scenarios was negative suggesting underestimation of the unobserved heterogeneity. The magnitude of bias for the frailty variance estimates were slightly higher when the sample size was 300 compared to the sample size of 500. Overall, the coverage probability of the frailty variance estimates consistently decreased as the known unobserved heterogeneity variance increased from 0.25 to 0.75. The coverage probability across all the models was slightly above 95% for scenarios where the unobserved heterogeneity variance was 0.25, suggesting some negligible over-coverage. For example, for scenario 1, coverage (MCSE) was 96.40 (0.5891), 95.90 (0.6270) and 96.20 (0.6046) for ISF-PH, LSF-NPHS1 and LSF-NPHS3 models respectively. Overall, substantial under-coverage was observed for scenarios where the unobserved heterogeneity variance was 0.50 or 0.75 among the flexible parametric models (LSF-NPHS1 and LSF-NPHS3). The under-coverage worsened as the sample size increased from 300 to 500. Such under-coverage suggested inaccuracy of the frailty variance estimates obtained using the LSF-NPHS1 and LSF-NPHS3 models.

As expected, overall, among the non-proportional hazard models with restricted cubic splines, the LSF-NPHS3 had lower bias and higher coverage of the frailty variance estimates compared to the LSF-NPHS1. This result suggests that for the non-proportional hazard models with restricted cubic splines, increasing the number of degrees of freedom modelling the baseline hazard function from 1 to 3 improves the frailty variance estimates. For the scenarios where the known unobserved heterogeneity was 0.50 or 0.75, we observed a higher magnitude of bias of frailty variance estimates for the non-proportional hazard models with restricted cubic splines models compared to the ISF-PH model. For the scenarios where the known unobserved heterogeneity variance was 0.25, we observed the lowest magnitude of bias for the frailty variance estimates in LSF-NPHS3 model followed by ISF-PH model then LSF-NPHS1 model.. For example, under scenario 1, the frailty variance estimate bias (MCSE) was − 0.0389 (0.0064), − 0.0556 (0.0060), − 0.0263 (0.0061) for ISF-PH, LSF-NPHS1 and LSF-NPHS3 models respectively. Pertaining to coverage, although the coverage probability decreased with increase in unobserved heterogeneity variance, decreasing the sample size from 500 to 300 yielded results with higher coverage. The decrease in coverage probability for the frailty variance estimates with increased unobserved heterogeneity was more drastic among the non-proportional hazards with restricted cubic splines models (LSF-NPHS1 or LSF-NPHS3) compared to the ISF-PH model. Overall, the ISF-PH model consistently yielded coverage probability closer to the nominal level of 95% compared to the non-proportional hazards with restricted cubic splines models (LSF-NPHS1 or LSF-NPHS3).

Overall, the MSE for the ISF-PH model frailty variance estimates were lower compared to the LSF-NPHS1 and LSF-NPHS3 models, except when the known unobserved heterogeneity variance was 0.25. Across all the 18 scenarios studied, we observed that the MSE estimates for the ISF-PH model decreased as the unobserved heterogeneity variance increased. However, the MSE estimates for the LSF-NPHS1 and LSF-NPHS1 models increased as the unobserved heterogeneity variance increased: Notably, the MSE for the LSF-NPHS3 model yielded lower MSE estimates compared to the LSF-NPHS1 model. As expected, increasing the sample size from 300 to 500 reduced the MSE estimates for the corresponding scenarios.

**Fig. 1:**
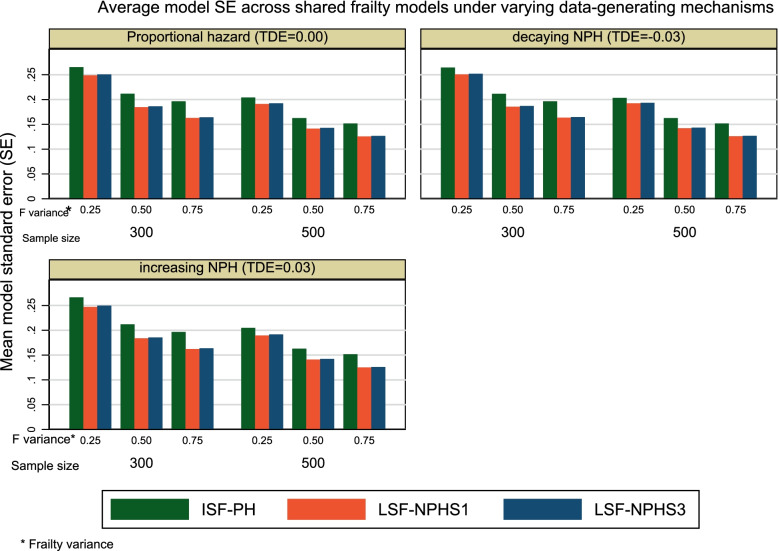
Average model standard errors (SE) for the frailty variance estimates across shared frailty models under varying data-generating mechanisms

Our investigation showed that LSF-NPHS1 and LSF-NPHS3 models were more efficient than the ISF-PH model as demonstrated by the lower mean of model-based standard errors (Fig. 1). The difference in model-based standard errors between that LSF-NPHS1 and LSF-NPHS3 models was negligible. As expected, increasing sample size from 300 to 500 improved the efficiency of the shared frailty models under study such that the model-based standard errors for the frailty variance estimates decreased.

### Percentage gain in precision for frailty variance estimates of non-proportional hazards shared frailty models with restricted cubic splines relative to ISF-PH model

Overall, as the unobserved heterogeneity increased, percentage gain in precision for frailty variance estimates of non-proportional hazards shared frailty models with restricted cubic splines relative ISF-PH model also increased (Table 1). The gain in precision was higher when time-dependent treatment effects were increasing (i.e. TDE = 0.03) compared to the decaying time-dependent treatment effects (TDE = -0.03). Across the models, the gain in precision was higher in the LSF-NPHS1 model compared to LSF-NPHS3 model. The observed gain in precision for LSF-NPHS1 and LSF-NPHS3 models compared to ISF-PH model under proportional hazard scenarios (i.e. TDE = 0) was similar to the scenarios when time-dependent treatment effects were increasing (i.e. TDE = 0.03).

### Bias and MSE for the estimated log hazard ratio estimates across the shared frailty models under varying unobserved heterogeneity and sample size

Overall, across all the three models under study, the MSE for the HR estimates increased as the unobserved heterogeneity variance increased (Table 2). The ISF-PH model consistently yielded lowest MSE estimates for the HRs across the three models regardless of the nature of the time-dependent treatment effect. Increasing the sample size from 300 to 500 also led to reduction in the estimated MSE for the HRs. We observed a loss in precision of HR estimates for the ISF-PH model compared to the LSF-NPHS1 and LSF-NPHS3 model. The precision loss ranged from 9 to 20%. The highest precision loss was observed when the unobserved heterogeneity was low (i.e. frailty variance equal to 0.25). The precision loss estimates were similar for both LSF-NPHS1 and LSF-NPHS3 models. There was low precision loss in the absence of time-dependent treatment effects (TDE = 0). In the presence of time-dependent treatment effects (i.e. TDE = 0.03 and TDE = -0.03), we observed higher magnitude of bias in the ISF-PH model compared to the LSF-NPHS1 and LSF-NPHS3 models. In the absence of the time-dependent treatment effects, the bias for the HR estimates was lower in the ISF-PH model compared to the LSF-NPHS1 and LSF-NPHS3 models. However, in the absence of the time-dependent treatment effects, the bias across all the three models was relatively lower (i.e. under 0.01) compared to scenarios when time-dependent effects are present.

**Table 2:** Bias and mean square error (MSE) for log hazard ratio estimates, estimated at 3 months, across shared frailty models in the presence of both unobserved heterogeneity and non-proportional hazards

Data Generating Mechanisms				ISF-PH Model	LSF-NPHS1 Model	LSF-NPHS3 Model	% gain in precision relative to ISF-PH model	
Scenario^a^	Sample size	Frailty variance	TDE	Bias (MCSE), MSE (MCSE)	Bias (MCSE), MSE (MCSE)	Bias (MCSE), MSE (MCSE)	LSF-NPHS1% gain in precision (MCSE)	LSF-NPHS3% gain in precision (MCSE)
1	500	0.25	0.03	−0.0284 (0.0021), 0.0050 (0.0002)	− 0.0033 (0.0023), 0.0051 (0.0002)	0.0021 (0.0023), 0.0052 (0.0002)	−17.4231 (2.1208)	− 18.7145 (2.0875)
2	500	0.50	0.03	− 0.0285 (0.0024), 0.0068 (0.0003)	− 0.0023 (0.0026), 0.0067 (0.0003)	0.0031 (0.0026), 0.0068 (0.0003)	−11.1578 (1.9985)	−12.3537 (1.9639)
3	500	0.75	0.03	−0.0233 (0.0027), 0.0076 (0.0004)	0.0035 (0.0029), 0.0081 (0.0004)	0.0093 (0.0029), 0.0083 (0.0004)	−12.7599 (1.9452)	−14.1747 (1.8756)
4	300	0.25	0.03	− 0.0259 (0.0028), 0.0085 (0.0004)	− 0.0015 (0.0030), 0.0093 (0.0004)	0.0041 (0.0031), 0.0096 (0.0004)	− 15.4391 (2.0856)	− 18.0137 (2.0061)
5	300	0.50	0.03	− 0.0215 (0.0032), 0.0105 (0.0005)	0.0047 (0.0033), 0.0111 (0.0005)	0.0103 (0.0034), 0.0114 (0.0005)	−9.4606 (2.1449)	−11.4682 (2.0577)
6	300	0.75	0.03	−0.0252 (0.0035), 0.0126 (0.0006)	0.0019 (0.0037) 0.0134 (0.0006)	0.0069 (0.0037), 0.0137 (0.0006)	− 10.3721 (1.9615)	− 12.1647 (1.8949)
7	500	0.25	−0.03	0.0287 (0.0021), 0.0051 (0.0002)	− 0.0075 (0.0023), 0.0053 (0.0002)	0.0018 (0.0023), 0.0053 (0.0002)	−18.3401 (2.1438)	−19.5151 (2.1016)
8	500	0.50	−0.03	0.0289 (0.0024), 0.0068 (0.0003),	− 0.0072 (0.0026), 0.0069 (0.0003)	0.0021 (0.0026), 0.0069 (0.0003)	−12.1807 (2.0392)	− 13.6435 (1.9931)
9	500	0.75	−0.03	0.0341 (0.0027), 0.0083 (0.0004)	−0.0011 (0.0029), 0.0083 (0.0004)	0.0086 (0.0029), 0.0085 (0.0004)	−14.1771 (1.9844)	−15.7796 (1.9005)
10	300	0.25	−0.03	0.0311 (0.0028), 0.0088 (0.0004)	− 0.0059 (0.0031), 0.0095 (0.0004)	0.0035 (0.0031), 0.0098(0.0004)	−16.7676 (2.1182)	−19.2818 (2.0272)
11	300	0.50	−0.03	0.0361 (0.0032), 0.0114 (0.0005)	0.0004 (0.0033), 0.0112 (0.0005)	0.0096 (0.0034), 0.0116 (0.0005)	−10.0998 (2.1977)	−12.4175 (2.0889)
12	300	0.75	−0.03	0.0316 (0.0035), 0.0130 (0.0006)	− 0.0032 (0.0037), 0.0136 (0.0006)	0.0052 (0.0037), 0.0139 (0.0007)	−11.6808 (2.0013)	− 13.7017 (1.9235)
13	500	0.25	0	−0.0009 (0.0021), 0.0042 (0.0002)	− 0.0051 (0.0023), 0.0052(0.0002)	0.0020 (0.0023), 0.0053 (0.0002)	−17.8005 (2.1303)	−19.0529 (2.0932)
14	500	0.50	0	−0.0009 (0.0024), 0.0059 (0.0003)	− 0.0044 (0.0026), 0.0067 (0.0003)	0.0027(0.0026), 0.0068 (0.0003)	−11.4262 (2.0238)	−12.6627 (1.9860)
15	500	0.75	0	0.0044 (0.0027), 0.0071 (0.0003)	0.0016 (0.0029), 0.0082 (0.0004)	0.0091 (0.0029), 0.0084 (0.0004)	−13.4164 (1.9621)	−14.9156 (1.8853)
16	300	0.25	0	0.0017 (0.0028), 0.0079 (0.0004)	−0.0032 (0.0031), 0.0094 (0.0004)	0.0041 (0.0031), 0.0097 (0.0004)	−15.8830 (2.0985)	−18.3944 (2.0120)
17	300	0.50	0	0.0061 (0.0032), 0.0101 (0.0005)	0.0027 (0.0033), 0.0112 (0.0005)	0.0097 (0.0034), 0.0115 (0.0005)	−9.7878 (2.1647)	−11.9642 (2.0672)
18	300	0.75	0	0.0021 (0.0035), 0.0120 (0.0006)	−0.0004 (0.0037), 0.0134 (0.0006)	0.0062 (0.0037), 0.0138 (0.0006)	−11.0009 (1.9807)	−12.9017 (1.9108)

### Example data application: Chloroquine for malaria in pregnancy trial

We illustrated the application of the three investigated shared frailty models above (ISF-PH, LSF-NPHS1 and LSF-NPHS3) to data on recurrent AEs collected from a clinical trial evaluating antimalarial drugs to prevent malaria in pregnancy. The trial is called *Chloroquine for malaria in pregnancy*.

The current modelling focussed on investigating the effect of treatment on recurrent AEs. We included a population of 600 pregnant women in their first or second trimesters who were randomized to receive IPTp of (a) sulfadoxine-pyrimethamine (SP, two doses, 4 weeks apart), (b) chloroquine (CQ, 600 mg on day 1, 600 mg on day 2, and 300 mg on day 3 two days apart). Although the original trial had three treatment arms, in the current analysis, we dropped the arm of CQ prophylaxis (to focus on IPTp arms) since its treatment schedule was radically different from the IPTp arms. Full details of the trial are published elsewhere [30].

Of the 600 pregnant women analysed in the current analysis, 474 (79%) experienced at least one AE. Cumulatively, more AEs were experienced in the CQ arm (895 AEs) than those in the SP arm (650 AEs). We fitted ISF-PH model followed by the LSF-NPHS1 and LSF-NPHS3 models where IPTp treatment was an exposure of interest. The SP treatment was considered as a reference treatment in the interpretation of the results. ISF-PH assumed the baseline hazard function followed Weibull distribution, unobserved heterogeneity followed inverse Gaussian distribution and the hazard rates between the CQ and SP treatments were proportional over the follow-up time. The non-proportional hazard models with restricted models (NPHS1 and LSF-NPHS3) assumed that unobserved heterogeneity followed lognormal distribution and time-dependent effects could be modelled using one degree of freedom. NPHS1 and LSF-NPHS3 approximated the baseline hazard function using 1 and 2 degrees of freedom respectively. We fitted adjusted and unadjusted model for all the three model under investigation. The adjusted model included the following covariates: treatment, maternal age, maternal body mass index and gravidity (i.e. whether the pregnant woman had previous pregnancy or not). We adjusted for the same covariates for the three models to ensure that the estimates were comparable. Since the results from the adjusted and unadjusted models were similar, our results reported in Table 3 below are based the unadjusted models since we considered them more parsimonious. The reported hazard ratios across the three models are based on marginal hazard ratio, predicted hazard ratio at 14 days and 60 days of follow-up. Additionally, we plotted the hazard ratio estimates over the follow-up time.

**Table 3:** Comparing sharing frailty models for analysis of recurrent AEs among pregnant women on IPTp treatment in Malawi

Estimate	ISF-PH	LSF-NPHS1	LSF-NPHS2^a^	LSF-NPHS3	LSF-NPHS4^b^
Log HR at 14 days (SE)	0.2281 (0.0499)	0.4758 (0.3572)	0.4343 (0.3724)	0.4648 (0.4033)	0.4404 (0.3710)
log HR at 60 days (SE)	0.2281 (0.0499)	0.0236 (0.1240)	0.2011 (0.2562)	0.2251 (0.2831)	0.1837 (0.2441)
Marginal log HR (SE)	0.2281 (0.0499)	0.2328 (0.0517)	0.3090 (0.0769)	0.3360 (0.0850)	0.3024 (0.0746)
Frailty variance	0.0324	0.0476	0.4591	0.6440	0.3977
AIC	6562.57	19,922.40	19,658.30	19,651.20	19,570.70

^a^Lognormal shared frailty model with non-proportional hazard and restricted cubic splines where the baseline hazard is modelled with 2 degrees of freedom and the time-dependent treatment effects is modelled with 1 degree of freedom

^b^Lognormal shared frailty model with non-proportional hazard and restricted cubic splines where the baseline hazard is modelled with 4 degrees of freedom and the time-dependent treatment effects is modelled with 1 degree of freedom

In this clinical example for recurrent AEs data analysis, the estimated marginal log hazard ratios were 0.2281 (0.0499)0.2328(SE: 0.0517), 0.3090 (0.0769), 0.3360 (SE:0.0850) and 0.3024(0.0746) for ISF-PH, LSF-NPHS1, LSF-NPHS2, LSF-NPHS3 and LSF-NPHS4 models respectively, indicating an increased risk of AEs among the women who took the CQ treatment arm compared to those in the SP treatment arm (Table 3). At 14 days, the Log HR across the non-proportional hazard models were similar ranging between 0.43 to 0.47 and were higher compared to the ISF-PH model. However, at 60 days only LSF-NPHS2, LSF-NPHS3 and yielded similar log HR estimates approximately 0.20 and were lower compared to the ISF-PH model estimates. The frailty variance estimates generally higher among the non-proportional hazard models compared to the ISF-PH model. Among the non-proportional hazards models with restricted cubic splines, the AIC estimates show that the model fit was good when the baseline hazard function was approximated with minimum of 2 degrees.

## Discussion

The analysis of recurrent AEs in clinical trials rarely account for potential unobserved heterogeneity and time-dependent effects [2]. Using a simulation study, we investigated the performance of three statistical methods for recurrent events that can be adapted towards the analysis of recurrent AEs in clinical trials, in the presence of both time-dependent effects and unobserved heterogeneity. Our paper mainly focused on evaluating the ability of the flexible parametric shared frailty models with non-proportional hazards and restricted cubic splines to capture both the unobserved heterogeneity and time-dependent treatment effects. We established that the inverse Gaussian shared frailty with proportional hazard model consistently yielded good coverage closer to the nominal level of 95%, lower bias and lower MSE of the frailty variance estimates at the expense of imprecision compared to the flexible shared frailty models with non-proportional hazards and restricted cubic splines. Interestingly, the frailty variance estimates coverage and bias, for the shared frailty models with non-proportional hazards and restricted cubic splines, improved upon increasing the number of degrees of freedom used to model the baseline hazard function. As expected, the flexible parametric shared frailty model with non-proportional hazards and restricted cubic splines yielded better hazard ratio estimates, reflecting the time-dependent treatment effects, compared to the inverse Gaussian shared frailty with proportional hazard model. The flexible parametric shared frailty models with non-proportional hazards and restricted cubic splines yielded unbiased estimates of HRs at the expense of imprecision and inaccuracy (i.e. high MSE) compared to the ISF-PH model. Therefore, our findings show that the more precise estimators were biased and imprecise estimators were unbiased. In drug safety assessment using methods that yield an unbiased hazard ratio would be of primary interest such that the flexible parametric shared frailty model should be considered if there is the presence of both unobserved heterogeneity and time-dependent effects. However, such a decision should be made at the cost of imprecision and loss of accuracy. Hence, we emphasize that the flexible shared frailty with non-proportional hazards and restricted cubic splines should be applied only when the proportional hazards assumption is violated. If interest is in estimating frailty variance, the inverse Gaussian model with proportional hazards can be considered sufficient even in the presence of both time-dependent treatment effect and unobserved heterogeneity.

The inverse Gaussian shared frailty with proportional hazard probably effectively captured the unobserved heterogeneity because we did not mis-specify the baseline hazard (i.e. Weibull distribution). Secondly, the longer follow-up period of 12 months made the population more homogeneous with time since the unobserved heterogeneity for inverse Gaussian frailty decays over the follow-up time [31]. Thirdly, the inverse Gaussian frailty is practically close to the lognormal frailty where the simulated data arose. The structure of the inverse Gaussian frailty would be considered more appropriate in the context of recurrent AEs in antimalarial drug trials where there are more frequent AEs early in the clinical trial [18]. Unfortunately, the conditional hazard ratio derived from the inverse Gaussian shared frailty with proportional hazard is constant over the follow-up time which does not reflect the non-proportionality of the hazards in the presence of time-dependent treatment effect.

The interpretation of our results is confined to scenarios where the censoring is assumed to be non-informative. In the presence of informative censoring, it will be interesting to understand how the models perform. Failure to account for time to medication presents a limitation in all the models considered in this work. Another key limitation of our study is relying on the assumption of constant unobserved heterogeneity. Future work can consider evaluating the performance of the shared frailty models accounting for both actual time to medication and time to AE occurrence under complicated scenarios where there is both time-varying treatment effects and time-varying unobserved heterogeneity.

Generally, in drug clinical trials, understanding drug safety profile requires efficient modelling of the recurrent AEs including accounting for both unobserved heterogeneity and any non-linear effects of the investigated drug. The methods studied in this paper provide key knowledge on choosing the most appropriate model for analysing recurrent AE data presenting the unobserved heterogeneity and time-dependent effects issues. The flexible parametric shared methods accounting for both time-dependent effects and unobserved heterogeneity are directly useful in IPTp trials since physiological changes that occur during pregnancy are mostly unobserved and can contribute to the heterogeneity of the treatment effect.

Based on our findings, we provide some practical recommendations on the studied shared frailty models to analyse the recurrent AEs when the hazards are non-proportional and the potential unobserved heterogeneity. Overall, the choice of the most appropriate model should be driven by the objective of the analysis. For instance, if interest is mainly in estimating hazard ratios, a shared frailty model with non-proportional hazards and restricted cubic splines is appropriate. When interest is in obtaining the unobserved heterogeneity estimates, inverse Gaussian with proportional hazard can serve the purpose. In the absence of time-dependent effects, using a shared frailty model with non-proportional hazards and restricted cubic splines should be avoided since it leads to biased estimates due to model overfitting.

## Data Availability

The code for simulated data generated is available from the corresponding author upon reasonable request. The *Chloroquine for Malaria in Pregnancy* (NCT01443130) trial data that support the findings of this study are available from Blantyre Malaria Project, Malawi but restrictions apply to the availability of these data, which were used under license for the current study, and so are not publicly available. Data are however available from the corresponding author upon reasonable request and with permission of Blantyre Malaria Project, Blantyre, Malawi.

## Abbreviations

IPTp: Intermittent preventive treatment for malaria prevention during pregnancy
AEs: Adverse events
RCTs: Randomized controlled trials
ISF-PH: Gaussian shared frailty model with proportional hazard
LSF-NPH1: Lognormal Shared frailty model with non-proportional hazards and single restricted cubic spline
LSF-NPH3: Lognormal Shared frailty model with non-proportional hazards and three restricted cubic splines
DGM: Data generating mechanism
TDE: Time-dependent effects
MCSE: Monte-Carlo Standard error
CQ: Chloroquine
SP: Sulfadoxine-pyrimethamine
